# Introduction of a remifentanil-based analgo-sedation protocol leads to a reduction of duration of mechanical ventilation and ICU stay in critically ill patients

**DOI:** 10.1186/cc9781

**Published:** 2011-03-11

**Authors:** J Van den Bosch, J Van Bommel, J Bakker, D Gommers

**Affiliations:** 1 Erasmus MC, Rotterdam, the Netherlands

## Introduction

Conventional sedation strategies in the ICU are based on the use of propofol or benzodiazepines for sedation in combination with morphine or other opioids for analgesia. An alternative strategy is based on analgo-sedation with remifentanil, a potent and very short-acting opioid agent. However, evidence is scarce that such a strategy is more efficacious.

## Methods

In January 2010 we introduced a remifentanil-based analgo-sedation protocol in our 32-bed academic general ICU. To evaluate the efficacy, we performed a retrospective comparison of all patients admitted between 1 April and 30 June 2010 with a control group consisting of patients admitted between 1 February and 30 September 2009 who underwent a conventional sedation strategy. Exclusion criteria were mechanical ventilation <24 hours, brain trauma, any other neurologic pathology, and moribund.

## Results

In total, 596 patients were selected in the conventional group (C) and 214 in the remifentanil group (R); after exclusion, group C consisted of 163 patients and group R of 70 patients for analysis. Both groups were identical in age, sex and APACHE II score. The mean duration of mechanical ventilation was significantly lower in group R (*P *= 0.01); time to successful detubation was significantly shorter in group R (log-rank *P *= 0.0026, HR = 0.57 (0.40 to 0.82). Overall ICU stay was shorter in group R; time to discharge to the ward was shorter in group R as well (log-rank *P *= 0.01, HR = 0.63 (0.44 to 0.90). ICU and hospital mortality as well as overall hospital stay were comparable in both groups.

## Conclusions

Introduction of a remifentanil-based analgo-sedation protocol significantly decreased duration of ventilation and ICU stay, most probably due to its short half-time, the easy titration of sedation and the absence of prolonged oversedation in critically ill patients.

